# Do COVID-19 Vaccinations Affect the Most Common Post-COVID Symptoms? Initial Data from the STOP-COVID Register–12-Month Follow-Up

**DOI:** 10.3390/v15061370

**Published:** 2023-06-13

**Authors:** Mateusz Babicki, Joanna Kapusta, Karolina Pieniawska-Śmiech, Żaneta Kałuzińska-Kołat, Damian Kołat, Agnieszka Mastalerz-Migas, Piotr Jankowski, Michał Chudzik

**Affiliations:** 1 Department of Family Medicine, Wroclaw Medical University, 51-141 Wroclaw, Poland; agnieszka.mastalerz-migas@umw.edu.pl; 2 Department of Internal Diseases, Rehabilitation and Physical Medicine, Medical University of Lodz, 90-647 Lodz, Poland; joanna.kapusta@umed.lodz.pl; 3 Department of Immunology and Pediatrics, The J. Gromkowski Provincial Specialist Hospital, 51-149 Wroclaw, Poland; karolina.pieniawska-smiech@student.umw.edu.pl; 4 Department of Clinical Immunology, Wroclaw Medical University, 50-368 Wroclaw, Poland; 5 Department of Experimental Surgery, Medical University of Lodz, 90-136 Lodz, Poland; zaneta.kaluzinska@umed.lodz.pl (Ż.K.-K.); damian.kolat@umed.lodz.pl (D.K.); 6 Boruta Medical Center, 95-100 Zgierz, Poland; 7 Department of Internal Medicine and Geriatric Cardiology, Medical Centre for Postgraduate Education, 01-813 Warsaw, Poland; piotrjankowski@interia.pl (P.J.); michalchudzik@wp.pl (M.C.); 8 Department of Nephrology, Hypertension and Family Medicine, Medical University of Lodz, 90-549 Lodz, Poland

**Keywords:** COVID-19, long COVID, COVID-19 vaccination, post-COVID, persistent symptoms, vaccine effectiveness

## Abstract

Around the world, various vaccines have been developed to prevent the SARS-CoV-2 virus infection and consequently the COVID-19 disease. However, many patients continue to report persistent symptoms after the acute phase. Since gathering scientific information on long COVID and post-COVID syndrome has become an urgent issue, we decided to investigate them in relation to the vaccination status of patients from the STOP-COVID registry. In this retrospective study, we analyzed data from the medical visit after contraction of COVID-19 and follow-up visits in the 3rd and 12th month after the disease. In total, 801 patients were included in the analysis. The most frequent complaints after 12 months included deterioration of exercise tolerance (37.5%), fatigue (36.3%), and memory/concentration difficulties (36.3%). In total, 119 patients declared that they had been diagnosed with at least one new chronic disease since the end of isolation, and 10.6% required hospitalization. The analysis of individual symptoms revealed that headache (*p* = 0.001), arthralgia (*p* = 0.032), and dysregulation of hypertension (*p* = 0.030) were more common in unvaccinated patients. Considering headache and muscle pain, people vaccinated after the disease manifested these symptoms less frequently. Subsequent research is needed to consider vaccines as a preventive factor for post-COVID syndrome.

## 1. Introduction

The outbreak of the COVID-19 pandemic in 2020, caused by the SARS-CoV-2 coronavirus, has led to over 6.8 million deaths worldwide [1,2]. The emergence of the deadly virus prompted a rapid reaction from scientists in the search for measures to limit this pathogen. In a short time, various types of vaccines were developed and subsequently delivered via mass distribution. As of March 2023, more than 13 billion doses of the COVID-19 vaccine have been administered worldwide [3], over 57 million of which were administered in Poland [4]. The vaccines were initially viewed with skepticism by the public due to rushed production; however, they have proven effective, significantly reducing the risk of developing severe and critical illness, as well as mortality caused by COVID-19 [1,5].

Although the struggle against the disease is not yet over and new cases of infection are still being recorded, the world is facing another crisis caused by the development and persistence of symptoms after the acute phase of COVID-19 called, depending on the duration of symptoms, long COVID or post-COVID syndrome [1]. According to the WHO, the post-COVID condition (also known as long COVID) is defined as “the continuation or development of new symptoms 3 months after the initial SARS-CoV-2 infection, with these symptoms lasting for at least 2 months with no other explanation” [6]. The National Institute for Health and Care Excellence’s definition of long-COVID includes new or ongoing symptoms 4 to 12 weeks after the acute phase of the illness is diagnosed. Additionally, this definition distinguishes between “ongoing COVID,” which is between 4 and 12 weeks, and “post-COVID syndrome,” for symptoms lasting more than 12 weeks [7].

Typical symptoms of post-COVID syndrome include fatigue, shortness of breath, cognitive and mental disorders such as anxiety and depression, chest pain, headache, balance disorders, insomnia, smell/taste disorders, joint pain, muscle pain, and palpitations [8]. At the population level, it is crucial to quantify the long-term burden of COVID-19 to assess its impact on the healthcare system and to identify risk factors of disease [1]. Older age, female gender, pre-existing comorbidities, and an acute course of COVID-19 are listed as the main risk factors for post-COVID in the literature [5].

Studies have shown that from 10% to 55% of patients after infection with the SARS-CoV-2 virus suffer from persistent symptoms [5] for weeks, months, and even several years [3,9]. This problem affects a wide spectrum of patients, from those who had no symptoms or experienced a mild course of the acute phase of COVID-19, to the seriously ill ones that require hospitalization and even treatment in intensive care units [8].

Two hypotheses are proposed regarding potential mechanisms to reduce the risk of developing post-COVID in previously vaccinated individuals. The first assumes that vaccines, by reducing the severity of the acute phase of SARS-CoV-2 infection, may reduce the risk of developing systemic and organ disorders, which ultimately may result in a reduced risk of symptoms and/or a reduction in the duration of symptoms. The second is based on the theory that vaccines may accelerate the elimination of the remaining coronavirus in the human body or reduce the excessive inflammatory and/or immune response associated with the development of post-COVID [3].

However, as can be observed, the impact of COVID-19 vaccination on the occurrence of long-term symptoms of the disease is not entirely clear [10]. Therefore, we undertook this study to assess whether COVID-19 vaccinations affect the most common post-COVID symptoms. The analyzed data concern the Polish STOP-COVID registry in the 12-month observation of patients.

## 2. Materials and Methods

This is a retrospective study based on patient data from the STOP-COVID registry, the largest Polish patient registry monitoring the health of people after COVID-19 (ClinicalTrials.gov identifier–NCT05018052). The STOP-COVID registry includes patients living in Poland who have contracted COVID-19 and who have been examined during a personal medical visit after contracting COVID-19 and follow-up visits in the 3rd and 12th month after the end of the disease.

The inclusion criteria for the program include:

(a) COVID-19 disease (confirmed by PCR or antigen tests in accordance with applicable regulations in the European Union);
(b) Age ≥ 18 years;
(c) Written consent to participate in the study.

Prior to the start of participation in the project, patients received information about the goals and methodology and then provided their informed consent to participate in the study. As part of medical visits, patients were subjected to a full physical and medical examination. In addition, within the first visit, data on socioeconomic status (including age, sex, and health status) were collected from the patient alongside assessment of chronic diseases such as hypertension, diabetes, hyperlipidemia, thyroid diseases, asthma and Chronic Obstructive Pulmonary Disease (COPD). Subsequently, the patient completed a questionnaire regarding the clinical symptoms they had during the COVID-19 infection. After the introduction of vaccination against COVID-19, information from patients on vaccination status was also collected. Subsequently, patients reported for a follow-up visit in the 3rd and 12th month. At subsequent visits, patients re-filled a questionnaire to assess the occurrence of symptoms in the 3rd and 12th month after COVID-19, respectively. The occurrence of the following symptoms was analyzed: chronic fatigue after infection, significant deterioration in exercise tolerance, smell and taste disorders, hair loss, skin lesions, memory and/or concentration disorders, headaches, muscle and joint pains, persistent cough, chest pain, fast/irregular heartbeat, and shortness of breath. Symptoms were selected from the most common ailments of the post-COVID syndrome [11]. Post-COVID was diagnosed according to the WHO definition [6]. At this stage, data on the status of COVID-19 vaccinations were supplemented. A vaccinated person was considered to be one who had received at least the basic vaccination schedule, i.e., 2 doses of Comirnaty (Pfizer/BioNTech), 2 doses of Spikevax (Moderna), 2 doses of Vaxzevria (AstraZeneca), or 1 dose of Johnson & Johnson. The timing of vaccination against COVID-19 (before or after COVID-19) was also evaluated. During the visit, whether the patient had been hospitalized for a reason other than COVID-19 in the last 12 months and whether a new chronic disease had been diagnosed during this period was also assessed. This study was conducted in accordance with the Declaration of Helsinki and the approval of the Bioethics Committee of Wroclaw Medical University was obtained.

### Statistical Analysis

The analysis was carried out using Statistica 13.0 by StatSoft. The analyzed variables were qualitative and quantitative. Basic descriptive statistics were used to describe the study group and the prevalence of post-COVID. The Shapiro–Wilk test was employed to assess the normality of the distribution. The chi-squared test was exploited to compare qualitative variables, and non-parametric tests (Mann–Whitney or Kruskal–Wallis) were used for quantitative variables. For examples in which statistically significant differences were demonstrated, a post hoc test (the Bonferroni test) was performed. A *p*-value < 0.05 was assumed to be statistically significant.

## 3. Results

**Figure 1:**
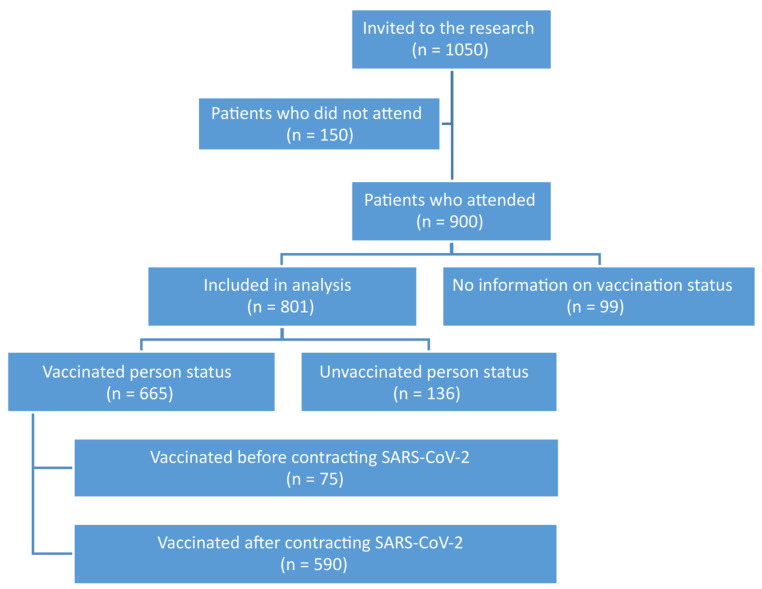
Characteristics of the study group.

In total, 1050 patients were enrolled for the follow-up visit in the 12th month, of which 150 did not attend. Of the data obtained from 900 patients, 99 did not provide or did not consent to the disclosure of information regarding the status of vaccination against COVID-19. Therefore, 801 patients were included in the final analysis, of which 665 (83.0%) were vaccinated. Analysis of the date of vaccination against the first COVID-19 infection indicated that 75 people (9.4%) were vaccinated before the disease and 590 (73.6%) after infection with SARS-CoV-2. The characteristics of the study group are presented in Figure 1. The vaccination status of the study group is summarized in Table 1.

Among the 801 patients, the vast majority were women (65.4%), and the average age of patients was 53.5 ± 12.8. The most common chronic diseases of patients during COVID-19 included hypertension (41.7%), hypercholesterolemia (19.9%), and thyroid diseases (16.5%). There were no differences in the distribution of sex, age, and chronic diseases between vaccinated and unvaccinated patients. This relationship was also not observed when assessing the timing of vaccination against COVID-19 (Table 2).

Among all patients presenting for follow-up after 12 months, 526 (65.7%) still declared the presence of at least one of the analyzed clinical symptoms. The most common complaints included deterioration in exercise tolerance (37.5%), fatigue (36.3%), and difficulties with memory and concentration (36.3%). In addition, 21.7% of patients reported hair loss, and 23.3% had experienced heart palpitations since COVID-19. Out of all patients, 119 people declared that they had been diagnosed with at least one new chronic disease since the end of isolation, and 10.6% required hospitalization during this time.

Analysis of the impact of vaccinations showed no differences between vaccinated and unvaccinated patients in the context of post-COVID syndrome diagnosis. However, when analyzing individual symptoms, it was shown that headache (*p* = 0.001), arthralgia (*p* = 0.032), and dysregulation of hypertension (*p* = 0.030) were significantly more common in unvaccinated subjects. Table 3 presents a detailed summary.

In analysis of the time vaccination against SARS-CoV-2 infection was obtained, both in the case of headache and arthralgia, people who were vaccinated after the disease manifested the above symptoms much less frequently. Post hoc analyses showed a difference between an unvaccinated and vaccinated person before COVID-19 (*p* = 0.031) and an unvaccinated and vaccinated person after COVID-19 (*p* < 0.001). A detailed breakdown is shown in Table 4 and Table 5.

## 4. Discussion

Post-COVID syndrome is currently attracting the interest of researchers around the world. Although it is a relatively new disease entity, the data from several years of observation can be found in a review of the literature. Nevertheless, researchers agree on the epidemiological threat of post-COVID syndrome. Despite many efforts, the causes of the syndrome are still not fully understood, and many questions remain unanswered. Similarly, the effect of vaccination on the risk of developing the disease could not be unequivocally assessed. 

In our study, among patients reporting for follow-up after 12 months, 65.7% (n = 526) declared the presence of at least one of the analyzed clinical symptoms, including deterioration in exercise tolerance (37.5%), fatigue (36.3%), and difficulties with memory and concentration (36.3%). A similar frequency of remaining symptoms was reported in a study conducted in France, where approximately 65% of patients after COVID-19 reported that at least one symptom persisted two months after the initial infection [12]. In the study published by Kim et al., where the observation time was 12 months, 48.8% of patients a year after being ill with COVID-19 still complained of symptoms, including memory deterioration (24.1%), insomnia (14.7%), fatigue (13.5%), and anxiety (12.9%) [13]. In a study by Pazukhina et al., post-COVID symptoms were present in 50% of adults 6 months after the disease and in 34% 12 months after [14].

The reasons for the discrepancies in the results of the cited studies are certainly multifactorial. Among them, aspects such as the study population (ethnic group, age structure, the issue of hospitalization of patients in the acute infection phase), the method of obtaining data, and the time elapsed from the acute infection to the analysis of symptoms should be considered.

The risk factors for post-COVID syndrome currently include female gender, older age, higher BMI, pre-existing comorbidities, smoking, previous hospitalization, and admission to an intensive care unit [15]. Risk factors have been quite well defined so far, but the data on protective factors, including the impact of vaccinations and the timing of their use on the occurrence of post-COVID, are scarce. Due to the growing scale of the problem, it began to be intensively explored, but the results of the studies are still inconsistent.

In a systematic review by Notarte et al., some studies indicated a reduction in persistent symptoms, while others indicated a slight change or even worsening of the patients’ condition [3]. In contrast, Nehme et al. observed that although vaccinations did not protect patients against post-COVID, they significantly reduced specific symptoms, such as fatigue, concentration problems, memory impairment, dyspnea, headache, and olfactory or taste disorders [16]. Similarly, there were no differences between vaccinated and unvaccinated patients in the context of post-COVID syndrome diagnosis in our study. However, when analyzing individual symptoms, it was shown that headache (*p* = 0.001), arthralgia (*p* = 0.032), and dysregulation of hypertension (*p* = 0.030) were significantly more common in unvaccinated subjects.

Some studies have provided strong evidence for vaccination as a method of long COVID prevention. Arnold et al. conducted a study involving hospitalized patients who developed long-term COVID-19. Researchers observed a reduction in the severity of persistent symptoms after COVID-19 in vaccinated patients [17]. When assessing the impact of vaccination on the risk of post-COVID development, it can be suspected that not only the fact of vaccination itself, but also the time of its administration may be important. In a study by Mizrahi et al., patients vaccinated prior to infection had a lower risk of prolonged dyspnea 30–90 days after COVID-19; however, these patients had a similar risk of other conditions compared to pre-infection unvaccinated patients [18]. Similarly, in the work of Al-Aly et al., the risk of post-COVID symptoms was lower in people who were vaccinated before SARS-CoV-2 infection compared to unvaccinated people [19]. However, the authors concluded that pre-infection vaccination provided only partial protection in the post-acute phase; therefore, relying on it as the only method of preventing post-COVID syndrome may not reduce the long-term health consequences of SARS-CoV-2 infection in an optimal way, which is also consistent with the results of our work. In a systematic review by Byambasuren et al., most of the included studies revealed a reduction in the persistence of symptoms after COVID-19 in patients who received a pre-infection vaccination, which is in line with supportive observational data and consistent with other beneficial effects of vaccination. It should be noted that studies included in the review had some limitations, including being based on ICD-10 codes rather than on self-reported symptoms [20,21]. Moreover, in a systematic review by Notarte et al., it has been shown that vaccination before infection can reduce the risk of post-COVID, though the strength of the evidence was considered to be low [3]. Interestingly, preliminary evidence suggests that receiving two doses of the vaccine is more effective than a single dose [3,22].

Interpretation of studies analyzing the impact of vaccination received after COVID-19 on the risk of post-COVID is even more difficult. While some studies show a positive effect of vaccination on the persistence of post-COVID symptoms, others have found no difference in this regard. Ayoubkhani et al. noted a 13% reduction in the development of long-term COVID-19 symptoms after the first dose of the vaccine, while a sustained improvement was observed after the second dose (with an average follow-up of 67 days) [23]. In a study by Richard et al., previously unvaccinated patients who were vaccinated after infection had a lower risk of persisting symptoms at the 6th month after infection, but no such difference was observed at the 12th month [24]. As with other studies, patients with higher initial severity of disease (moderate to severe) were more likely to report persistent symptoms than those with mild initial symptoms. Contradictory results were obtained in the study by Wisnivesky et al., where 453 patients with at least one post-COVID symptom were described, of whom 73% subsequently received the vaccine. After six months, there was no difference in post-COVID symptoms between the vaccinated and unvaccinated groups; however, there were differences in the characteristics of the study and control groups at the beginning, which could have influenced the obtained results [8]. In our work, the studied group had comparable characteristics in terms of sex, age, and chronic diseases. In analysis of the time of obtaining vaccination, in relation to the acute phase of SARS-CoV-2 infection, people who were vaccinated after the disease were less likely to present with headache and myalgia.

Joint pain, along with fatigue, is a common post-COVID symptom. Pro-inflammatory reactions, characteristic for SARS-CoV-2 infection, can affect almost every system, including the musculoskeletal system. Musculoskeletal pain, fatigue, and decreased exercise tolerance are some of the typical musculoskeletal sequelae [25]. Synovial cells, including fibroblasts, monocytes, B cells, and T cells, show ACE2 and TMPRSS2 expression. Joint cartilage has ACE2 receptors, which have also been found in osteoblast-rich tissues. The presence of these receptors suggests that skeletal muscle, synovium, and cortical bone may serve as potential sites for direct infection with SARS-CoV-2 and its likely long-term sequelae [26]. Some cytokines and molecules, such as C-X-C motif chemokine 10, interferon-gamma, interleukin (IL)-1β, IL-6, IL-8, IL-17, and tumor necrosis factor-alpha, are induced by infection and play an important role in the pathogenesis of both acute and persistent sequences of symptoms associated with COVID-19. IL-1β and IL-6 can lead to fibrosis that results from increased muscle fibroblast activity [25]. Therefore, it seems interesting that, 12 months after documented SARS-CoV-2 infection, joint pain persists significantly more often among unvaccinated patients. Perhaps this is related to a stronger pro-inflammatory response in unvaccinated people compared to vaccinated people.

Despite the lack of sufficient evidence, hypertension is suspected to be a potential risk factor for post-COVID [27]. Hypertension and related conditions may be part of a sequence of symptoms that persist after COVID-19, as shown in several studies [28,29,30]. The exact pathophysiological mechanisms responsible for damage to the cardiovascular system in patients experiencing post-COVID syndrome are still not clearly defined, but potential causes include damage to the vascular endothelium or damage to cardiomyocytes [31]. Most of the studies examining the correlation between post-COVID syndrome and hypertension were conducted in the early phase of the pandemic, and only a small number of studies have considered new variants of coronavirus or included vaccinated patients. In our study, dysregulation of previously controlled and treated hypertension was significantly more common in unvaccinated subjects, which may have been due to less organ damage in the acute phase of infection in previously vaccinated patients. However, further research and observations are needed in this regard.

The authors are aware of the limitations of this study. Undoubtedly, the main limitation is the selection of the study group. Patients who did not attend a follow-up visit in the 12th month and for whom no feedback was obtained regarding their health were excluded from the observation. One of the reasons for resignation from the visit may be the disappearance of symptoms, which undoubtedly may affect the final results of the observation. However, it should be emphasized that the authors attempted to contact the above persons, which turned out to be ineffective. It should also be mentioned that the analyzed group is not representative of Polish society. Another limitation is the lack of knowledge about the variant that the patient was infected with, the treatment used during the acute phase of COVID-19, or potential reinfections during the observation period, which may have a significant impact on the obtained results. Another limitation is the lack of differentiation regarding the severity of the acute phase of COVID-19. In addition, the coexistence of other infections, such as Epstein–Barr virus (EBV) and hepatitis C virus (HCV), was not analyzed. Finally, the lack of knowledge about the specific COVID-19 vaccine used by the patient is also a downside of our research.

On the other hand, it should be noted that the literature lacks data on the impact of COVID-19 vaccination on post-COVID syndrome. In addition, the innovation of our study is also the differentiation of patients according to whether they had been vaccinated before or after SARS-CoV-2 infection. Despite the methodological limitations of the study, according to the authors, this research provides preliminary evidence that COVID-19 vaccination may have an impact on the type of symptoms manifested in post-COVID syndrome. Undoubtedly, this topic requires further deepening of understanding and that a study be conducted on a large, representative group after excluding all the previously mentioned limitations.

Since post-COVID is a relatively new disease entity with a position that is yet to be established, it is necessary to collect as much research data as possible to assess the prevalence of disease symptoms and factors that may affect their occurrence, especially those of a modifiable nature. Vaccination against COVID-19 is certainly such a factor, and its impact may also depend on the time elapsed between SARS-CoV-2 infection and the assessment of persistent symptoms.

## 5. Conclusions

Post-COVID is a significant health issue, and persistent symptoms can last for months. The results of our study provide evidence that COVID-19 vaccination is an important element in the prevention of some symptoms belonging to post-COVID syndrome in the long term. The performed analyses demonstrate that vaccination has significantly contributed to reducing the prevalence of headaches in post-COVID syndrome. However, there is not enough evidence to consider it as a preventive factor for post-COVID syndrome as a whole, and this is a phenomenon that requires further observation and extensive research on representative groups of patients.

## Figures and Tables

**Table 1:** Vaccination status.

N (%)	
Vaccination status	
Vaccinated	665 (83.0)
Unvaccinated	136 (17.0)
Date of vaccination against COVID-19	
Before COVID-19	75 (9.4)
After COVID-19	590 (73.6)
Unvaccinated	136 (17.0)

**Table 2:** Characteristics of the study group including vaccination status.

Variable		Whole Group [N (%)]	Vaccination Status [N (%)]			Vaccination Time [N (%)]			
			Vaccinated	Unvaccinated	*p*	Before COVID-19	After COVID-19	Unvaccinated	*p*
Age [M ± SD]		53.5 ± 12.8	52.0 ± 13.7	53.9 ± 12.6	0.178	55.9 ± 14.1	52.0 ± 13.6	53.9 ± 12.6	0.176
Sex	Woman	524 (65.4)	437 (83.4)	87 (16.6)	0.696	59 (11.3)	378 (72.1)	87 (16.6)	0.051
	Man	277 (34.6)	228 (82.3)	49 (17.7)		16 (5.8)	212 (76.5)	49 (17.7)	
Chronic diseases	Hypertension	354 (41.7)	280 (83.8)	54 (16.2)	0.605	39 (11.6)	241 (72.2)	54 (16.2)	0.161
	Hypercholesterolemia	159 (19.9)	131 (82.4)	28 (17.6)	0.812	115 (72.3)	16 (10.1)	28 (17.6)	0.905
	Diabetes	81 (10.1)	69 (85.2)	12 (14.8)	0.584	61 (75.3)	8 (9.9)	12 (14.8)	0.858
	Asthma	87 (10.9)	67 (77.0)	20 (23.0)	0.113	59 (67.8)	8 (9.2)	20 (23.0)	0.282
	COPD	15 (1.9)	12 (80.0)	3 (20.0)	0.753	11 (73.3)	1 (6.7)	3 (20.0)	0.904
	Thyroid diseases	132 (16.5)	111 (84.1)	21 (15.9)	0.720	97 (73.5)	14 (10.6)	21 (15.9)	0.832

M–mean; SD–standard deviation.

**Table 3:** The clinical picture of patients 12 months after the end of COVID-19 for the whole group and concerning vaccination status.

	Whole Group [N (%)]	Vaccination Status		Size Effect	*p*
		Vaccinated	Unvaccinated		
At least one symptom	526 (65.7)	435 (65.4)	91 (66.9)	0.011	0.737
Fatigue	291 (36.3)	240 (36.1)	51 (37.5)	0.011	0.755
Worse tolerance for exercise	300 (37.5)	248 (37.3)	52 (38.2)	0.007	0.836
Taste and olfactory dysfunction	62 (7.7)	50 (7.5)	12 (8.8)	0.018	0.603
Hair loss	174 (21.7)	139 (20.9)	35 (25.7)	0.044	0.212
Skin lesions	40 (5.0)	32 (4.8)	8 (5.9)	0.018	0.602
Excessive sweating	134 (16.7)	111 (16.7)	23 (16.9)	0.002	0.950
Headache	156 (19.5)	116 (17.4)	40 (29.4)	0.113	0.001
Memory and concentration problems	291 (36.3)	239 (35.9)	52 (38.2)	0.018	0.612
Arthralgia	50 (6.2)	36 (5.4)	14 (10.3)	0.075	0.032
Myalgia	144 (18.0)	121 (18.2)	23 (16.9)	0.012	0.722
Palpitations	187 (23.3)	157 (23.6)	30 (22.1)	0.013	0.696
Peripheral edema	70 (8.7)	55 (8.3)	15 (11.0)	0.036	0.299
Newly diagnosed arterial hypertension	102 (12.7)	77 (11.6)	25 (18.4)	0.077	0.030
Fainting/unconsciousness	16 (2.0)	16 (2.4)	0 (0.0)	0.065	0.136
Dyspnea	113 (14.1)	92 (13.8)	21 (15.4)	0.017	0.623
Cough	85 (10.6)	72 (10.8)	13 (9.6)	0.015	0.661
Chest pain	119 (14.9)	100 (15.0)	19 (14.0)	0.012	0.749
New chronic disease within 12 months from COVID-19 ending (N = 794)	119 (14.9)	101 (15.3)	18 (13.3)	0.021	0.555
Hospitalization due to chronic disease (n = 798)	85 (10.6)	73 (11.0)	12 (8.8)	0.027	0.448

Significant differences (*p* < 0.05) were marked with bold characters.

**Table 4:** The clinical picture of patients 12 months after the end of COVID-19 concerning the time of vaccination against SARS-CoV-2 infection.

	Vaccination Status			Size Effect	*p*
	Before COVID-19	After COVID-19	Unvaccinated		
At least one symptom	47 (62.7)	388 (65.8)	91 (66.9)	0.022	0.821
Fatigue	29 (38.7)	211 (35.8)	51 (37.5)	0.021	0.843
Worse tolerance for exercise	28 (37.3)	220 (37.3)	52 (38.2)	0.007	0.973
Taste and olfactory dysfunction	5 (6.7)	45 (7.6)	12 (8.8)	0.210	0.847
Hair loss	19 (25.3)	120 (20.3)	35 (25.7)	0.056	0.282
Skin lesions	5 (6.7)	27 (4.6)	8 (5.9)	0.033	0.642
Excessive sweating	12 (16.0)	99 (16.8)	23 (16.9)	0.006	0.983
Headache	20 (26.7)	96 (16.3)	40 (29.4)	0.137	**<0.001**
Memory and concentration problems	26 (34.7)	213 (36.1)	52 (38.2)	0.020	0.853
Arthralgia	17 (22.7)	19 (3.2)	14 (10.3)	0.244	**<0.001**
Myalgia	16 (21.3)	105 (17.8)	23 (16.9)	0.029	0.707
Palpitations	13 (17.3)	144 (24.4)	30 (22.1)	0.051	0.365
Peripheral edema	7 (9.3)	48 (8.1)	15 (11.0)	0.038	0.549
Newly diagnosed arterial hypertension	10 (13.3)	67 (11.4)	25 (18.4)	0.785	0.084
Fainting/unconsciousness	2 (2.7)	14 (2.4)	0 (0.0)	0.0648	0.189
Dyspnea	12 (16.0)	80 (13.6)	21 (15.4)	0.026	0.752
Cough	5 (6.7)	67 (11.4)	13 (9.6)	0.046	0.421
Chest pain	9 (12.0)	91 (15.4)	19 (14.0)	0.029	0.698
New chronic disease within 12 months from COVID-19 ending (n = 794)	7 (9.5)	66 (11.2)	12 (14.1)	0.031	0.673
Hospitalization due to chronic disease (n = 798)	5 (6.8)	96 (16.4)	18 (13.3)	0.081	0.076

Significant differences (*p* < 0.05) were marked with bold characters.

**Table 5:** Post hoc analysis for headache and arthralgia.

Vaccination	Vaccination before COVID-19	Vaccination after COVID-19	Unvaccinated
**Headache**			
Vaccination before COVID-19	–	0.032	<0.001
Vaccination after COVID-19	0.032	–	<0.001
Unvaccinated	<0.001	<0.001	–
**Arthralgy**			
Vaccination before COVID-19	–	<0.001	0.031
Vaccination after COVID-19	<0.001	–	<0.001
Unvaccinated	0.031	<0.001	–

## Data Availability

The data presented in this study are available on request from the corresponding author.
